# Titanium dioxide nanoparticles promote arrhythmias via a direct interaction with rat cardiac tissue

**DOI:** 10.1186/s12989-014-0063-3

**Published:** 2014-12-09

**Authors:** Monia Savi, Stefano Rossi, Leonardo Bocchi, Laura Gennaccaro, Francesca Cacciani, Alessio Perotti, Davide Amidani, Rossella Alinovi, Matteo Goldoni, Irene Aliatis, Pier Paolo Lottici, Danilo Bersani, Marco Campanini, Silvana Pinelli, Marta Petyx, Caterina Frati, Andrea Gervasi, Konrad Urbanek, Federico Quaini, Annamaria Buschini, Donatella Stilli, Claudio Rivetti, Emilio Macchi, Antonio Mutti, Michele Miragoli, Massimiliano Zaniboni

**Affiliations:** Department of Life Sciences, University of Parma, Parma, Italy; Department of Clinical and Experimental Medicine, University of Parma, Parma, Italy; CERT, Center of Excellence for Toxicological Research, Department of Clinical and Experimental Medicine, Via Gramsci 14, Parma, 43126 Italy; Department of Physics and Earth Science, University of Parma, Parma, Italy; National Research Council- IMEM-CNR, Parma, Italy; Italian Worker Compensation Authority INAIL, ex-ISPESL Monteporzio Catone, Roma, Italy; Department of Biomedical, Biotechnological and Translational Sciences (S.Bi.Bi.T), University of Parma, Parma, Italy; Department of Pharmacology, Second University of Naples, Naples, Italy; Humanitas Clinical and Research Center, Via Manzoni 56, Rozzano, Milan 20090 Italy

**Keywords:** Pollution, Cardiac arrhythmia, Experimental model, Titanium dioxide nanoparticles, Supernormal conduction, Membrane leakage

## Abstract

**Background:**

In light of recent developments in nanotechnologies, interest is growing to better comprehend the interaction of nanoparticles with body tissues, in particular within the cardiovascular system. Attention has recently focused on the link between environmental pollution and cardiovascular diseases. Nanoparticles <50 nm in size are known to pass the alveolar–pulmonary barrier, enter into bloodstream and induce inflammation, but the direct pathogenic mechanisms still need to be evaluated. We thus focused our attention on titanium dioxide (TiO_2_) nanoparticles, the most diffuse nanomaterial in polluted environments and one generally considered inert for the human body.

**Methods:**

We conducted functional studies on isolated adult rat cardiomyocytes exposed acutely *in vitro* to TiO_2_ and on healthy rats administered a single dose of 2 mg/Kg TiO_2_ NPs via the trachea. Transmission electron microscopy was used to verify the actual presence of TiO_2_ nanoparticles within cardiac tissue, toxicological assays were used to assess lipid peroxidation and DNA tissue damage, and an *in silico* method was used to model the effect on action potential.

**Results:**

Ventricular myocytes exposed in vitro to TiO_2_ had significantly reduced action potential duration, impairment of sarcomere shortening and decreased stability of resting membrane potential. *In vivo*, a single intra-tracheal administration of saline solution containing TiO_2_ nanoparticles increased cardiac conduction velocity and tissue excitability, resulting in an enhanced propensity for inducible arrhythmias. Computational modeling of ventricular action potential indicated that a membrane leakage could account for the nanoparticle-induced effects measured on real cardiomyocytes.

**Conclusions:**

Acute exposure to TiO_2_ nanoparticles acutely alters cardiac excitability and increases the likelihood of arrhythmic events.

**Electronic supplementary material:**

The online version of this article (doi:10.1186/s12989-014-0063-3) contains supplementary material, which is available to authorized users.

## Background

The explosive growth in nanotechnology – i.e., the design and development of systems at the atomic or nano scale – and bioengineering nanotechniques has led to the development of a large number of new nanomaterials with novel biological, physical and chemical properties. Nanoparticles (NPs) have been manufactured for several decades on an industrial scale. Several metal oxide NPs possess photo-catalytic ability, high electrical conductivity, ultraviolet absorption and photo-oxidizing capacity against chemical and biological species [1]. Some commercial products, such as cosmetics, are also likely source of NPs that could become uncontrollably delivered to the human environment [1]. Because of the widespread presence of potential sources of NPs, environmental release of manufactured NPs is expected to increase for the near future. Thus, understanding the molecular and cellular basis of nanotoxicity is an essential challenge today.

We focused the present study on one of the most-produced NPs species, namely titanium dioxide (TiO_2_), 1.45 million metric tons of which were produced in the United States in 2007 alone [2], and which was considered biologically inert up to recently [3]. Most studies concerning TiO_2_-NP toxicology have focused to date on pulmonary inflammation [4-7] and have elucidated its behavior inside cells. Unfortunately, it has been very difficult to detect, track and precisely quantify TiO_2_-NPs at the cellular level. Thus, the effects on cellular and nuclear membrane protein domains, as well as on the interference with metabolic pathways, remain poorly investigated.

The National Institute of Occupation Safety and Health (NIOSH) recommends airborne exposure limits of 2.4 mg/m^3^ for fine TiO_2_ and 0.3 mg/m^3^ for ultrafine TiO_2_, as time-weighted average concentrations for up to 10 hours per day during a 40-hour work week, but NIOSH also admits that there is insufficient data to classify TiO_2_ as a hazard for human health [8,9].

Recently, attention has moved to the study of how TiO_2_ translocates from lungs to other systemic organs [1,10], including the gastrointestinal tract, kidney and heart [11]. Indeed, the heart is the first organ that inhaled TiO_2_-NPs may reach, after passing the alveolar epithelial fenestrated barrier and entering the pulmonary circulation. Thus, we hypothesized that acute exposure to TiO_2_-NPs might cause detrimental effects on cardiac electromechanical function. In fact, we have previously described in cultured engineered neonatal cardiac tissue that TiO_2_-NPs may influence cardiac action potential (AP) by enhancing conduction and upstroke velocities and disrupting myofibrils via production of reactive oxygen species (ROS) [12]. However, comprehensive *in vivo* assessment of cardiac risk is lacking.

Here, we demonstrate with conventional electrophysiological techniques – i.e., patch-clamp, Epicardial Potential Mapping (EPM) and cellular motion detection – that acute exposure (<5 hours) to TiO_2_-NPs (diameter range: 30–100 nm) is detrimental for cardiac performance and increases the propensity for arrhythmia. Biophysical characterization of the NPs was conducted with a number of techniques – i.e., Atomic Force Microscopy (AFM), Dynamic Light Scattering (DLS), Raman spectroscopy, and Transmission Electron Microscopy (TEM). TiO_2_ toxicology was also characterized with ROS and ThioBarbituric Acid Reactive Substance (TBARS) analyses.

## Results

### Particles size, type and aggregation

**Figure 1:**
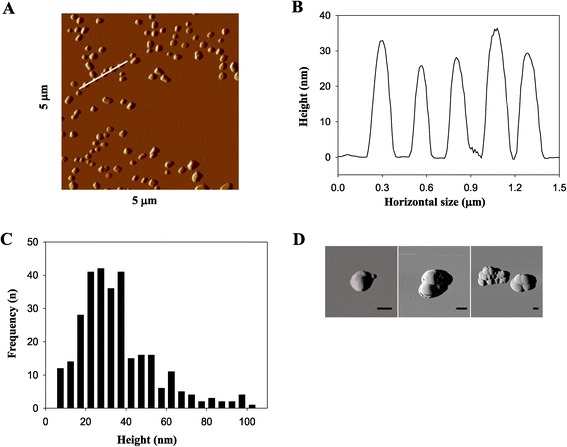
**Atomic Force Microscopy analysis of titanium dioxide nanoparticles (TiO**_**2**_ **-NPs) deposited on poly-ornithine-treated mica. A**. Image of deposed TiO_2_-NPs. **B**. Height profile along the white line shown in **A**. **C**. Height distribution of TiO_2_-NPs. **D**. Images of TiO_2_-NP aggregates (scale bars =100 nm).

AFM imaging revealed that a relevant fraction of TiO_2_-NPs had a diameter <100 nm (Figure 1A): specifically, single NPs had a diameter in the 25–35 nm range (Figure 1B); the overall size distribution frequency of the NPs is given in Figure 1C. In addition, NP aggregates of variable size and morphology were also present, composed of up to tens of single particles (Figure 1D). By measuring volume, we estimated that ~40% of NPs had a diameter <100 nm, with the remaining particulates made up of aggregates.

The Raman spectrum of the TiO_2_-NPs (Additional file 1: Figure S1) had peaks corresponding to a mixture of anatase (tetragonal polymorph, space group I4_1_/amd, characterized by Raman peaks at ~143, 196, 396, 516 and 638 cm^−1^) and rutile (tetragonal polymorph, P4_2_/mnm, with characteristic Raman frequencies at ~143, 238, 445 and 609 cm^−1^) TiO_2_ minerals. All peaks for TiO_2_-NPs were larger than those of the pure polymorphs, confirming the presence of nanosized (<100 nm) TiO_2_ particles [13,14]. The amount of anatase was determined with a calibration procedure using the intensities of the Raman peaks of the two polymorphs present in the mixture (see Additional file 1: Methods section). The results of this procedure on different Raman peaks coherently indicated 93 wt% anatase in the TiO_2_-NP powder, with an estimated uncertainty of about ±1%. Finally, in order to better characterized charge and size of the adopted NPs, DLS was employed: Z-potential and hydrodynamic diameter values are reported in Table 1.

**Table 1:** **Biophysical properties on TiO**_**2**_ **NPs in different solutions**

**TiO**_**2**_ **-NPs**	**Water**	**Saline**	**Tyrode**	**PBS-BSA**
Z-Potential (mV)	−31.74 ± 1.02	−18.36 ± 2.30	−24.60 ± 1.61	−13.05 ± 2.13
HydrodynamicDiameter (nm)	154 ± 3.00	498 ± 0.55	538 ± 0.37	449 ± 0.85

### TiO_2_ nanoparticles impair cardiomyocyte contractility

**Figure 2:**
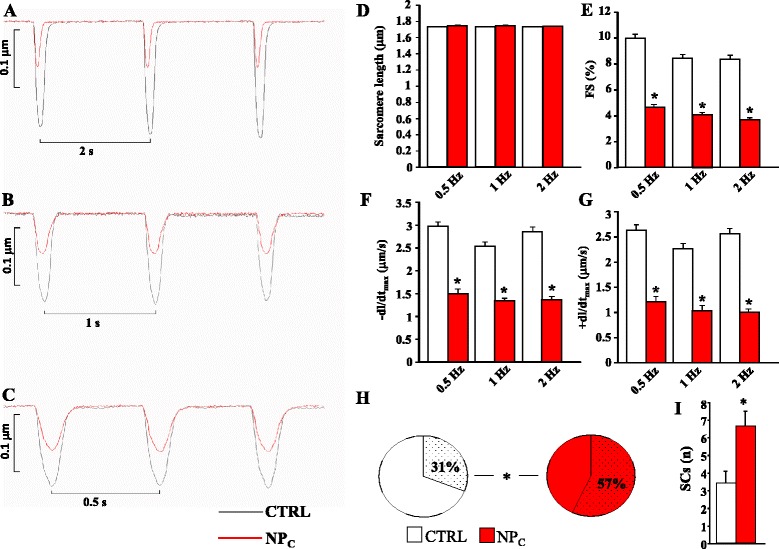
**Representative traces of sarcomere shortening recorded in CTRL (black) and NP**_**C**_ **(red) cardiomyocytes field-stimulated at 0.5 (A), 1 (B) and 2 (C) Hz.** Graphs of resting sarcomere length **(D)**, sarcomere fractional shortening (FS) **(E)**, maximal rate of shortening (−dl/dt_max_) **(F)**, and maximal rate of re-lengthening (+dl/dt_max_) **(G)**. **H**. Pie charts of the percentages of cardiomyocytes exhibiting spontaneous contractions (SCs, stippled areas) in CTRL (white) and NP_C_ (red) cells after 60 s of conditional training at 0.5 Hz. **I**. Graph of number of SCs/cardiomyocyte in the 60 s measurement period. *, p <0.01 vs. CTRL.

The effect of TiO_2_ on contractility was investigated on cardiomyocytes isolated from adult rats: n = 104 non-exposed control cardiomyocytes (CTRL), and n = 102 TiO_2_-NP-exposed cells (NP_C_). Figure 2A–C give representative recordings of shortening under different pacing frequencies (0.5 Hz, 1 Hz and 2 Hz) in CTRL (black line) and NP_C_ (red line). The average diastolic sarcomere length (Figure 2D) was nearly identical in CTRL and NP_C_ at all pacing rates (1.73 ± 0.003 μm and 1.74 ± 0.003 μm, respectively). In contrast, fractional shortening (FS) and maximal rates of shortening and re-lengthening were significantly reduced in NP_C_ at all frequencies (~ −50%, p < 0.01; Figure 2E–G).

Spontaneous contractions (SCs) were then assessed in cardiomyocytes that had been conditionally trained for 60 s with field stimulation set at 0.5 Hz. The percentage of cells exhibiting SCs in the 60 s after stopping electrical pacing was significantly higher in NP_C_ than in CTRL (Figure 2H). Furthermore, in cardiomyocytes exhibiting SCs, the number of events per cell within the 60 s period was two-fold higher in NP_C_ (Figure 2I, Additional file 1: Figure S2).

### TiO_2_ nanoparticles slightly depolarize resting membrane potential but dramatically alter other action potential parameters

**Figure 3:**
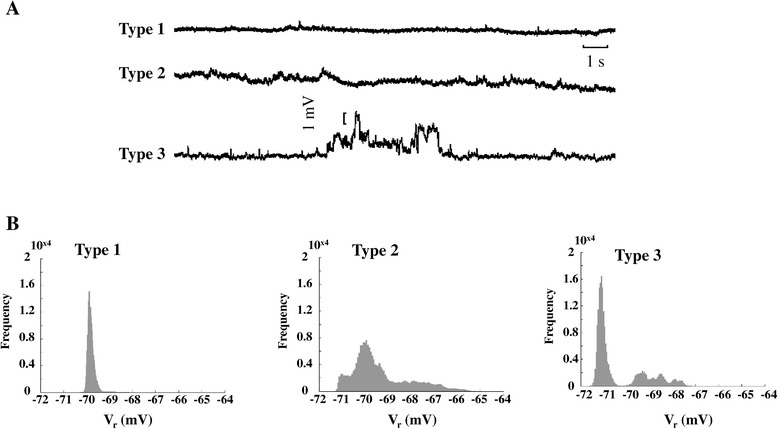
**Variability in resting membrane potential in cardiomyocytes exposed to TiO**_**2**_ **NPs. A**. Traces representative of the three types of V_r_ behavior found over a 60 s recording period subsequent to conditioning training at 5 Hz for 40 beats. **B**. Frequency distribution of V_r_ for the three ΔV_r_ types.

**Figure 4:**
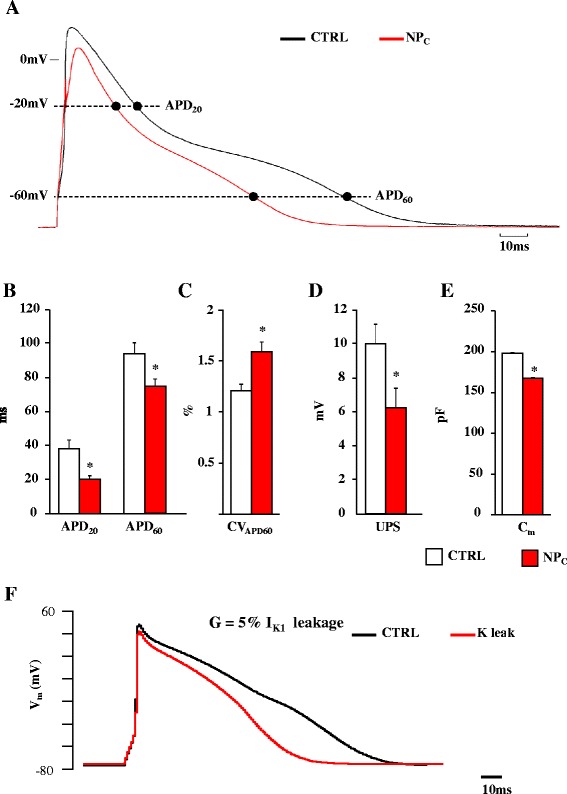
**TiO**_**2**_ **NPs-induced changes in cellular electrophysiology. A**. Representative action potential (AP) waveforms recorded from control (CTRL, black line) and TiO_2_-NP (NP_C_, red line) cardiomyocytes at the physiological driving rate of rat heart (5 Hz). **B–E**. Graphs of action potential duration (APD) measured at **−**20 mV (APD_20_) and **−**60 mV (APD_60_), beat**-**to**-**beat variability of APD_60_ (CV_APD60_), AP upstroke (UPS) and membrane capacitance (C_m_). In all graphs, CTRL is given by white columns, and NP_C_ by the red columns (n = 37 NP_C_ and n = 49 CTRL). *, p < 0.05 vs. CTRL. **F**. APs simulated with the Pandit model, without (black trace, CTRL) and with (red trace, NP_C_) a simulated 1.5 nS constant potassium leakage.

In order to assess whether SCs were associated with sub- or supra-threshold fluctuations of resting membrane potential (V_r_), we analyzed variability (ΔV_r_) over 60 s recording periods in the absence of electrical stimulation; similarly to motion measurements, cells were conditionally trained (40 beats at 5 Hz) before V_r_ recording. We found three types of V_r_ fluctuation (Figure 3A): in one group of cardiomyocytes (type 1), V_r_ was stable over time – considering the expected signal-to-noise ratio for this type of voltage recording – with a sharp frequency distribution (Figure 3B, left panel); in a second group (type 2), V_r_ underwent small (~1 mV), continuous fluctuations, resulting in a continuously distributed frequency around a peak value of −70 mV (Figure 3B, middle panel); finally, type 3 cardiomyocytes displayed dispersed transitions to discrete V_r_ levels (ΔV_r_ up to 3–4 mV), resulting in well-separated peaks within the frequency distribution (Figure 3B, right panel). The fraction of type 2 and 3 cardiomyocytes increased after exposure to TiO_2_-NPs (64% in NP_C_ vs. 36% in CTRL). Overall, ΔV_r_ and the coefficient of variability of V_r_ (CV_Vr_) were significantly higher in NP_C_ (ΔV_r_: 2.8 ± 0.24 mV in CTRL vs. 4.0 ± 0.35 mV in NP_C_; CV_Vr:_ 0.53 ± 0.04 in CTRL vs. 0.70 ± 0.07 in NP_C_). TiO_2_-NP-exposed cardiomyocytes also had, on average, a remarkable reduction in AP duration (APD) with respect to both the early (APD_20_) and late (APD_60_) phases of repolarization (Figure 4A,B). A dose-dependent effect was found at concentrations from 5 to 50 μg/ml (Additional file 1: Figure S3). Of note, despite APD shortening – which is expected to decrease beat-to-beat variability of APD in this cell type [15] – CV_APD60_ increased significantly by 28% (Figure 4C).

AP upstroke (UPS) and electrical membrane capacitance (C_m_) values were also significantly reduced after acute exposure to TiO_2_-NPs (Figure 4D–E). Mean values of V_r_, resting membrane resistance (R_m_), AP amplitude (APA), and rheobase and chronaxie values were comparable in NP_C_ and CTRL (Additional file 1: Table S1).

In order to provide a mechanistic explanation for the cellular findings, we ran simulations with a modified version of the Pandit et al. rat ventricle AP model [16]. The above-presented *in vitro* findings could be reproduced *in silico* by introducing into the Pandit model’s pool of ion currents a 1.5 nS leakage conductance selectively permeable to potassium ions (for the sake of comparison, amounting only to ~5% of maximum I_K1_ conductance). Of note, the experimental and simulated APs were similar, with a quasi-superimposable reduction of APD without any significant changes in V_r_ (compare Figures 4A and F; Additional file 1: Figure S4). Moreover, we ran simulations of Pandit-modelled APs with and without the addition of a K^+^ leakage current, and setting extracellular [K^+^] at values ranging from 3.0 to 23.2 mmol/l. We found that the simulated leakage current led to an increase in dV/dt_max_ from 180 to 183 V/s (Additional file 1: Figure S5, left panel), with a maximum peak corresponding to a [K]_o_ of about 6 mmol/l, which is known to characterize supernormal conduction of sodium current in engineered neonatal rat cardiac tissue [17] (Additional file 1: Figure S5, right panel).

### ECG and epicardial electrograms indicate faster electrical activation after exposure to TiO_2_

**Figure 5:**
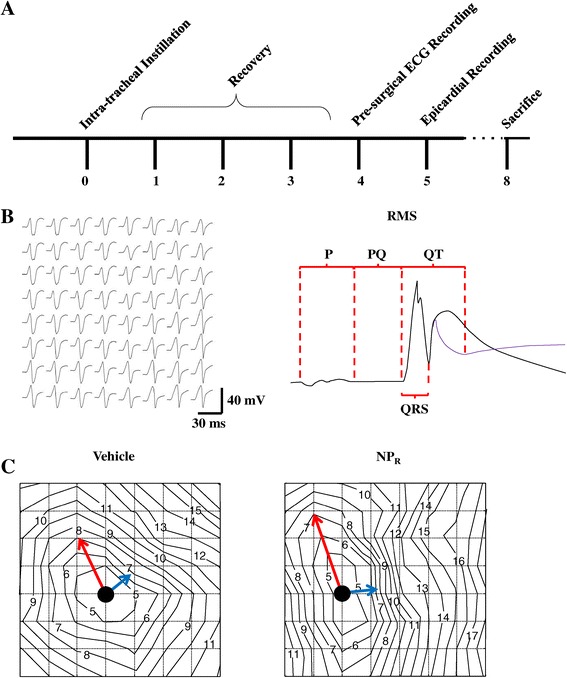
**Instillation of TiO**_**2**_ **-NPs and** ***in vivo*** **recordings of cardiac electrical performance. A**. Time-scale (hours) of the experimental protocol. **B**. Representative EGs recorded from an 8×8 epicardial electrode array. Each waveform of the grid represents the time-course of extracellular potential at the corresponding position. The scheme on the right hand side explains the EG parameters, as measured from their root mean square (RMS)-derived signals. Magenta thin trace represents the first time derivative, whose minimum value is taken as a marker of the end of the QT interval **C**. Representative activation time maps (isochrones, ms) from Vehicle (left) and NP_R_ (right), showing differences in longitudinal (red arrows) and transverse (blue arrows) propagation.

Rats were anesthetized as described below and instilled tracheally with either physiological solution (Vehicle) or 2 mg/Kg TiO_2_-NPs (NP_R_). Rats were left to recover for 4 hours before undergoing anesthesia (see Figure 5A). NP_R_ had a normal QRS pattern of activation, as evidenced by 3-lead ECG (data not shown). Electrograms (EGs) derived from the electrode array positioned on the epicardial surface (Additional file 1: Figure S6) did not indicate any qualitative difference in the two experimental groups. Indeed, EGs (Figure 5B) had the expected biphasic form, with positive electrocardiographic R and negative S waves whose amplitudes were correlated to the area explored by the electrode array. In detail, EGs had an Rs shape (see Additional file 1: Methods section) when positioned near the heel of the pulmonary artery, and an rS shape near breakthrough points.

Since EG waves were not qualitatively different, we focused our attention on their duration (right panel of Figure 5B, Table 2). We found that P wave duration and the PQ segment were significantly reduced (−12%), as was QRS complex duration (−5%), in NP_R_ compared with Vehicle. There was a non-significant increase in heart rate (R-R reduction), while QT duration was reduced 22% in NP_R_ (Table 2).

**Table 2:** In-vivo electrophysiological parameters

	**Vehicle**	**NP**_**R**_
P wave (ms)	33.0 ± 0.22	28.9 ± 0.21**
PQ segment (ms)	24.2 ± 0.18	21.3 ± 0.21**
QRS complex (ms)	16.0 ± 0.09	15.2 ± 0.09**
QT interval (ms)	40.0 ± 0.28	31.1 ± 0.54**
RR interval (ms)	237.6 ± 0.90	231.3 ± 2.47
Rheobase (μA)	21.4 ± 1.68	20.6 ± 2.73
Chronaxie (ms)	0.75 ± 0.04	0.64 ± 0.04*
CVI (m/s)	0.63 ± 0.004	0.70 ± 0.004
CVt (m/s)	0.33 ± 0.002	0.32 ± 0.002
Anisotropy ratio	1.96 ± 0.01	2.33 ± 0.02**

* p < 0.05 vs Vehicle **p < 0.005 vs Vehicle.

### Exposure to TiO_2_ nanoparticles increases cardiac excitability and conduction velocity

*In vivo*, we observed a non-significant decrement of rheobase (Vehicle: 21.4 ± 1.68 μA; NP_R_: 20.6 ± 2.73 μA) and significantly decreased chronaxie (Vehicle: 0.75 ± 0.04 ms; NP_R_: 0.64 ± 0.04 ms; p < 0.05) in NP_R_, pointing to an overall increase in cardiac excitability (Table 2).

From the isochrone maps, we evaluated the average transverse and longitudinal conduction velocity (CVt and CVl, respectively) (Figure 5C). While CVt was unchanged, CVl increased significantly by 11% in NP_R_ (Table 2, **, p < 0.005). The anisotropy ratio CVl/CVt – the augmentation of which is recognized as a key arrhythmogenic factor [18] – was 19% higher in NP_R_ (**, p < 0.005).

### Tracheal instillation of TiO_2_ nanoparticles exacerbates arrhythmogenesis

**Figure 6:**
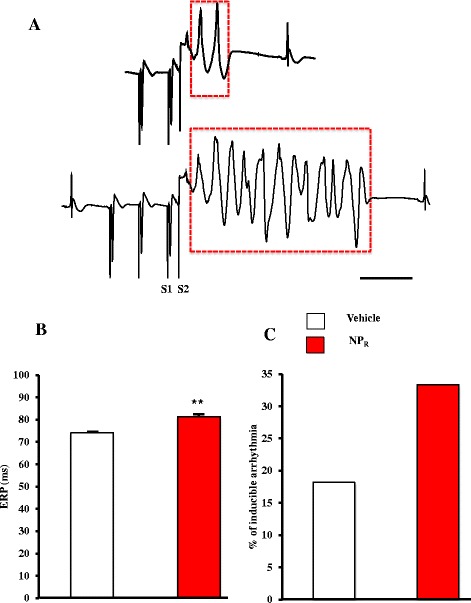
**Susceptibility to arrhythmias in Vehicle and NP**_**R**_ **rats. A**. Ventricular ectopic couplet (top) and ventricular fibrillation (bottom) recorded during evaluation of the effective refractory period (ERP). Scale bar =500 ms. **B**. Evaluation of ERP in Vehicle and NP_R_. **, p < 0.005. **C**. Percentage of inducible arrhythmc events.

Refractoriness was measured as the effective refractory period (ERP) using a dedicated protocol [19,20] (Figure 6) – see Additional file 1: Methods section - for parameters definition. An increase in ERP duration and ERP spatial dispersion – commonly recognized as an index of vulnerability to arrhythmia – was also found in NP_R_ (Figure 6B–C).

Similarly, the likelihood of establishing arrhythmic events during the ERP was assessed from 60 electrode positions in NP_R_ and 44 in Vehicle. We observed a roughly two-fold increase in induced ventricular ectopic activity, mainly extra-systole couplets (Figure 6A, top) and ventricular fibrillation (Figure 6A, bottom), when pacing near the ERP (33% of the total electrodes for NP_R_ vs. 18% Vehicle; Figure 6C).

### TiO_2_ nanoparticles acutely reach lungs and ventricles, and are internalized within cells

**Figure 7:**
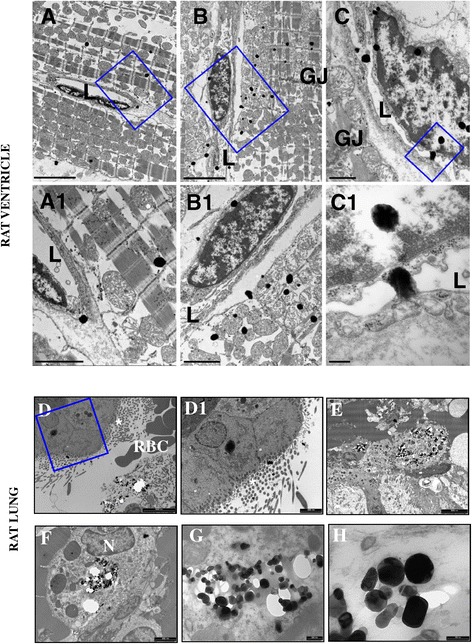
**Presence of titanium dioxide (TiO**_**2**_ **) nanoparticles (NPs) in the rat ventricular myocardium after tracheal instillation: TEM analysis. A**. Right Ventricle. Electron-dense NPs in two longitudinally oriented cardiomyocytes and in the wall of a vascular structure. **B**. Left Ventricle. NPs accumulating at the edge of longitudinally oriented cardiomyocytes, as well as in the sarcolemma. NPs are also present in the interstitial space, in endothelial cells and within the capillary lumen (L). **C**. Left ventricle. The lumen of a capillary neighboring a cardiomyocyte containing TiO_2_ NPs, which also appear to be connected to and engulfed by endothelial cells. GJ marks a gap junction location. Blue rectangles include areas shown at higher magnification in the lower panels (A1, B1 and C1). Scale Bars: A and B =5 μm; A1 and B1 = 2 μm; C =1 μm; C1 = 200 nm. Bottom. Ultrathin sections of lung samples from NP-exposed treated rats. **D**. The bronchial epithelium is apparent by the presence of ciliated cells (*). Electron-dense NPs are best seen in cytoplasm at high magnification (**D1**). Clusters of NPs were found within the lung parenchyma **(E)** and in macrophages **(F)**. N, nucleus. **G,H**. The typical shape of titanium NPs is apparent at higher magnification. Scale Bars: D =5 μm; D1 = 2 μm; E =2 μm; F =1 μm; G =200 nm; H =100 nm.

A key aspect for the interpretation of the above findings was to identify the presence of TiO_2_-NPs not only in the lungs but also in exposed cardiomyocytes. We found that TiO_2_-NPs entered not only directly into cultured cardiomyocytes (Additional file 1: Figure S7A,B), but also *in vivo* into left and right ventricular cardiomyocytes of TiO_2_-instilled rats, suggesting that contamination of cardiac tissue can occur via the lungs. In particular, morphologic evidence provide by TEM indicates that NPs leave the capillary lumen, cross the endothelial layer, penetrate the sarcolemma and reach the myoplasm by establishing intimate contact with myofibrils and mitochondria (Figure 7A–C). Ultrastructural analysis also showed the presence of TiO_2_-NPs in lung tissue, with a tendency to agglomerate in the cytoplasm of alveolar cells and macrophages (Figure 7D–H).

### TiO_2_ Nanoparticles are cardiotoxic and genotoxic

**Figure 8:**
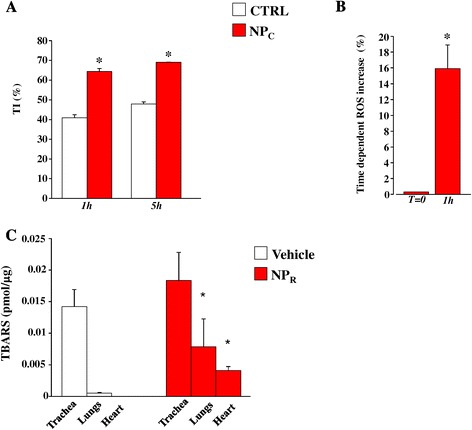
**TiO**_**2**_ **NPs-induced toxicological effects. A**. DNA damage detected in single isolated cardiomyocytes by Comet assay (pH >13) in CTRL (white columns) and NP_C_ (red columns) after 1 h and 5 h of exposure. DNA damage is expressed as tail intensity (TI%; *p < 0.05). **B**. Percent increase of ROS in single isolated cardiomyocytes, NP_C_ (red column) after 1 h. **C**. Evaluation of TBARS in trachea, lungs and heart tissue after tracheal instillation of saline solution (Vehicle) or saline solution containing TiO_2_-NPs (2 mg/Kg, NP_R_). *, p < 0.05 vs. Vehicle.

The interplay between TiO_2_-NPs and cardiomyocytes denoted signs of intracellular damage after acute administration. Indeed, we observed a 25% increase in damaged nuclear DNA already after one hour of exposure, accompanied by a 16% increase in ROS formation (Figure 8A, B). We also analyzed TBARS *ex vivo*, from trachea, lungs and heart. While only tracheal tissue showed positive TBARS values in Vehicle –probably owing to the instillation maneuver – NP_R_ had lipid peroxidation in the lungs as well as in the heart (Figure 8C), indicating membrane damage of cardiomyocytes due to ROS production.

## Discussion

Our findings indicate that TiO_2_-NPs can: i) reach the heart via the respiratory system; ii) enter ventricular cardiomyocytes; and iii) enhance the susceptibility to cardiac arrhythmias via shortening of repolarization time, and by increasing cardiac excitability. To the best of our knowledge, this is the first time that a direct effect of TiO_2_ on cardiac electrical performance has been reported and explained *in vivo*.

It was described recently that, depending on size and bioavailability, NPs may reach the bloodstream after passing the pulmonary barrier [10]. Furthermore, we previously reported that electrically charged NPs modulate excitability by producing membrane disruption (positively charged NPs) or life-compatible nanopores (negatively charged NPs) [21]. We also reported that negatively charged nanoparticles can be internalized via clathrin-mediated membrane protrusions [22]. Such findings are in line with our leakage hypothesis and explain our observation of a significant decrease in membrane capacitance due to membrane loss caused by the internalization process.

TiO_2_ is widely used as a nanomaterial because it is considered inert [23,24]: its toxicity, documented in cellular and animal models, remains controversial [25,26] since surface charge may differ depending on the vehicle [26]. Z-potential characterization demonstrated that NPs were negatively charged in our experimental setting and, therefore, able to transiently produce nanopores that, in turn, resulted in the development of leakage currents, as reported previously [21]. It is known that, upon tracheal instillation, TiO_2_ generates a detrimental sequence of dysfunctions, which might ultimately lead to atherosclerosis and, thus, affect the function of the heart pump [27]. Here, we show how quickly TiO_2_-NPs may directly affect cardiac electrical activity via the reduction of repolarization velocity, enhancing temporal dispersion and increasing excitability.

In order to test the hypothesis that an NP-induced leakage current might provide, *per se*, a mechanistic explanation of our electrical findings, we introduced a 1.5 nS potassium leakage into the membrane equations of the Pandit rat ventricular AP model [16]. Of note, simulations were consistent with the results obtained in patch-clamped cardiomyocytes, i.e., reduction of APD and UPS, and slight depolarization of V_r_. In order to provide a mechanistic explanation of the increment of CVl found *in vivo*, we simulated electrically paced AP trains by means of the Pandit model endowed or not with the leakage current. Simulated APs exhibited the highest ΔdV/dt_max_ for [K]_o_ =5.8 mmol/l, which corresponds to the point of supernormal sodium conduction [17] in cardiac tissue, suggesting that the small TiO_2_-induced depolarization may: i) speed up the kinetics for reaching the activating threshold, and ii) lead to the observed increment in CVl, which can thus be ascribed to supernormal conduction [12]. Notably, conduction block and increased likelihood of alternans [28] consequent to supernormal conduction are known to make cardiac tissue prone to structural and functional reentrant arrhythmias [17,29]. Although arrhythmogenesis is frequently associated with prolongation of repolarization, measured at the cellular and organ level (APD and QT), the role of QT and APD shortening in favoring transition to arrhythmic events has been also extensively documented [30]. This is particularly relevant for our findings, where TIO_2_-NPs – in contrast with diesel-exhaust particulates, for instance [31] – induced APD and QT shortening.

Also, physiological sarcomere contraction is undoubtedly affected by the presence of TiO_2_. In fact, not only were NPs detected among the myofibrils by TEM, but dynamic sarcomere measurements on the same cells indicated that their excitation–contraction coupling machinery was significantly impaired. Besides the reported arrhythmogenic behavior due to dyssynchronous sarcomere contractions mediated by the mechano-electric feedback [32,33], we also found an increased incidence of spontaneous contractions (cf. Figure 3) following high-frequency conditioning pacing, which may be synergic with an increment of inducible arrhythmic events.

Although our experiments do not reveal any evidence for NP-induced changes in rate-dependent shortening of the sarcomere, their lengths, FS and maximum rates of shortening and re-lengthening (±dl/dt_max_) were all affected by the presence of NPs. Our multi-level approach generated parallel *in vitro* and *in vivo* results (reduction in the APD, increase in the APD temporal dispersion, reduction in the QT interval and increase in its dispersion), which substantially rule out cardiac inflammatory process caused by TiO_2_-NPs as the main mechanism responsible for excitation–contraction coupling unbalance. Nonetheless, chronic effects, including inflammatory progression, certainly need further evaluation.

A recent study on Langendorff-perfused isolated heart elegantly demonstrated that TiO_2_-NPs produced abnormal electrical activity accompanied by a dose-dependent increase in heart rate [34]. Our study confirms those findings in *in vivo* anesthetized rats (ECGs and EGs) and focuses for the first time on the underlying mechanism. Indeed, the evaluation of excitability parameters *in vivo* indicated that cardiac tissue becomes more excitable, in line with previously described results [12]. Moreover, we adopted a single NP dose (2 mg/Kg) in solution in order to be aligned not only with NIOSH recommendations on the time-evaluation of human exposure to TiO_2_-emitting sources, but also because such a concentration in ~50 μl of instilled solution is a good compromise between the volume of vehicle needed and the optimal disaggregation of NPs (cf. Figure 1). A half-dose instillation of TiO_2_-NPs (1 mg/Kg) evaluated in three rats produced the same effects observed with the full dose (data not shown).

Although the pathological effects on the cardiovascular system are gaining more attention than they have had in the past [35], the effect of NPs on cardiovascular tissue is usually viewed as secondary to inflammatory processes initiated at the lungs [36]. Despite the fact that the latter mechanism can be present chronically, we observed in the acute setting that ROS production and an increase in inducible arrhythmias occurred after perfusion with TiO_2_-NPs, which were rapidly internalized within cardiomyocytes. ROS-induced membrane damage and NP-induced leakages are expected to locally depolarize cardiac tissue and promote arrhythmogenesis [37] via source-sink electrical mismatch. Occurrence of arrhythmias was estimated in healthy rat hearts *in vivo* by the well-known S1-S2 protocol (see Additional file 1: Methods section). Through this protocol, we found a significant increase in the incidence of ectopic events (duplets or triplets) and of ventricular fibrillation in rats administered TiO_2_. Therefore, these malignant events may be exaggerated by acute and sub-acute exposure to an environment polluted with TiO_2_.

### Study limitations

While this study proposes supernormal sodium-based conduction at the single-cell level as a physiological mechanism underlying the arrhythmia *in vivo*, it only partially clarifies the difference in anisotropy ratio observed in rats exposed to NPs. Further investigation is necessary to evaluate: i) the effect of TiO_2_-NPs on cell-to-cell coupling, e.g., intercalated discs; ii) the possible re-localization of connexins from the end to the side of sarcolemmal microdomains as responsible for ensuring longitudinal and lateral connection among cardiomyocytes; and iii) the possible pro-arrhythmic role of inflammatory processes in lungs, vasculature and myocardium. Moreover, chronic exposure to TiO_2_-NPs needs to be investigated in order to confirm not only the molecular mechanisms underlying the propensity to arrhythmias, but also to identify a time limit for workers who are exposed daily to TiO_2_.

## Conclusion

Our results clearly describe the short-term direct effect of TiO_2_-NPs on myocardial tissue. Although additional epidemiological and toxicological studies will be required to further establish a link, TiO_2_ exposure is very likely to increase propensity to arrhythmias, and thus needs to be monitored in terms of ultra-fine particle emissions and length of exposure (in the range of a few hours). Notably, our study highlights a rapid effect that is not based on cumulative NP absorption and that could possibly lead to a fast-developing pro-arrhythmic scenario.

## Methods

### Experimental animals

The study population consisted of male Wistar rats bred in our departmental animal facility, aged 12–14 weeks and weighing 300–350 g. The animals were kept in single-sex groups of four individuals from weaning (4 weeks after birth) until the onset of the experiments, in a temperature-controlled room at 20–24°C, with the light on between 7.00 AM and 7.00 PM. The bedding of the cages consisted of wood shavings; food and water were freely available. Rats were anesthetized with a mixture of 40 mg/kg ip ketamine chloride (Imalgene, Merial, Milano, Italy) and 0.15 mg/kg ip medetomidine hydrochloride (Domitor, Pfizer Italia S.r.l., Latina, Italy), both for the *in vivo* and *ex vivo* experiments. This study was carried out in accordance with the recommendations in the Guide for the Care and Use of Laboratory Animals of the National Institute of Health. The protocol was approved by the Veterinary Animal Care and Use Committee of the University of Parma and conforms to the National Ethical Guidelines of the Italian Ministry of Health (Permit number: 41/2009-B). All effort was made to minimize suffering.

### Particle suspension

TiO_2_-NPs (Titanium (IV) oxide, Sigma, code: 677469, Milan, Italy) were suspended in sterilized high**-**purity water at a concentration of 2.5 mg/ml for the *in vitro* studies, and in physiological saline solution (10 mg/ml, stock solution) for *in vivo* experiments. Immediately before the experiments, the suspensions were vortexed and directly sonicated (Branson Ultrasonics, Danbury, CT, USA) through five cycles of 20 s at 65% of the maximum power at room temperature in order to minimize particle aggregation.

### TiO_2_ nanoparticle characterization

After sonication, the TiO_2_-NP suspension at 2.5 mg/ml was diluted in low-calcium solution ([Ca^2+^] =0.1 mmol/l) to a final concentration of 50 μg/ml and analyzed by AFM. A drop of TiO_2_-NP suspension was deposed onto freshly cleaved mica and onto mica treated with poly-ornithine prepared as follows: 10 μl poly-ornithine solution (10 mg/ml) was deposed onto freshly cleaved mica for 1 minute, the disk was then rinsed with milliQ water and dried with a gentle nitrogen flow. Afterward, the NP suspension (50 μg/ml) was deposed and incubated for 5 minutes at room temperature. The mica disk was then rinsed with milliQ water and dried with nitrogen. AFM imaging was performed on the dried sample with a Nanoscope IIIa microscope equipped with scanner J and operating in tapping mode. Commercial diving board silicon cantilevers (MikroMasch, Tallinn, Estonia) were used. Images were analyzed with ImageJ software. The percentage of NPs <100 nm, with respect to the total number of NPs present in the topographical image was determined using the “Laplacian volume” routine of Gwyddion software (ver. 2.32, see Additional file 1: Methods section).

The anatase and rutile proportions of the TiO_2_ preparation used were determined with a Jobin-Yvon Labram micro-Raman apparatus equipped with an Olympus BH-4 confocal microscope with 4x, 10x, 50x and 100x objectives (lateral spatial resolution of approximately 25, 10, 2 and 1 μm, respectively). The spectrometer employs a 20 mW He-Ne Laser emitting at 632.8 nm, an edge filter, a 256×1024 pixel CCD detector, an 1800 grooves/mm grating and a density filter wheel. The spectral resolution is about 1 cm^−1^. The calibration of the spectrometer was controlled on the silicon Raman peak at 520.6 cm^−1^. Raman spectra were acquired with 4x and 10x objectives for 2–5 seconds and 5–8 repetitions, and recorded at 10 different points for each sample. The baseline subtraction with a 2nd degree polynomial curve, the normalization and the peak fitting were made with LABSPEC 5.78.24, Jobin Yvon/Horiba software package. Since the behavior and the aggregation state of NPs strongly depend on their surface charge and the ionic strength of the medium, we carried out further characterizations using DLS and Z-potential techniques. The measurements were performed using the 90Plus Phase Analysis Light Scattering (PALS) instrument by Brookhaven Corp. Particle size measurements were made with a 658 nm laser, collecting data at a scattering angle of 90°. In order to estimate the Stokes-Einstein hydrodynamic radius of the NP agglomerates in suspension, we performed a measurement of the autocorrelation function fitted, assuming a lognormal distribution of relaxation times. The measurement of the Z-potential was carried out by PALS, which determines the electrophoretic mobility of charged colloidal suspensions [38].

### Single-cell studies

#### Cardiomyocyte isolation

Individual myocytes were enzymatically isolated from the left ventricle (LV) with collagenase perfusion in accordance with a procedure previously described [15] (see Additional file 1: Methods section).

### Acute exposure of adult cardiomyocytes to TiO_2_ nanoparticles

Half of the freshly isolated cells were incubated with buffered maintenance solution (see Additional file 1: Methods section) supplemented with TiO_2_-NPs (stock solution of 2.5 mg/ml in high purity water) at final concentrations of 50, 25 and 5 μg/ml. Patch-clamp and contractility recordings were started 1 hour after exposure to TiO_2_-NPs, and lasted 2–5 hours. Oxidative stress and genotoxicity in single cells were assessed in the same experimental conditions.

### Contractility properties

Mechanical properties of CTRL and NP_C_ were assessed by using the IonOptix fluorescence and contractility systems (IonOptix, Milton, MA) as previously described [39]. Briefly, cells were field-stimulated at frequencies of 0.5, 1 or 2 Hz by constant depolarizing pulses (2 ms in duration, and twice diastolic threshold in intensity) by platinum electrodes placed on opposite sides of the cell chamber connected to a MyoPacer Field Stimulator (IonOptix). The stimulated cardiomyocytes were displayed on a computer monitor by means of the IonOptix MyoCam camera connected to the side port of the inverted microscope (Nikon TE2000, Nikon Instruments, Japan). The following parameters were computed: mean diastolic sarcomere length, FS (%) and ± dl/dt_max_. See Additional file 1: Methods section.

### Patch-clamp recordings

CTRL and NP_C_ in the whole-cell patch-clamp configuration were used for transmembrane potential (V_m_) measurements in current-clamp mode using a Multiclamp 700B amplifier (Molecular Devices, Sunnyvale, CA, USA ). Data recordings and analysis were performed with Clampfit9 software (Molecular Devices, Sunnyvale, CA, USA). Stability of V_m_ during resting conditions was assessed as ΔV_r_ (maximum–minimum V_r_ values, mV) and CV_Vr_ (%) over 60 s recordings. C_m_ and R_m_ were derived according to a protocol previously described [40]. Sequences of APs were elicited by means of brief (3 ms) depolarizing current pulses with amplitude 1.5 times the current threshold, and sampled at 10 kHz. From the average of 10 consecutive beats, AP parameters, i.e., UPS, APA and APD at −20 mV (APD_20_) and at −60 mV (APD_60_) were measured as described in the supplementary section.

### Action potential simulations

All simulations reported in this study were performed by means of the Pandit et al. rat ventricular AP model [16]. The model was recompiled in Matlab language using the COR facility at http://cor.physiol.ox.ac.uk. The “ode15S’ Solver built into the R2010b version of Matlab (MathWorks Inc., Navick, MA, USA) was used to integrate model equations. Simulations were run on a PC with an Intel Core 2.24 GHz CPU. Initial conditions for all simulations were set after a simulated train of 50 conditioning beats elicited at a frequency of 2 Hz. A leakage current (I_leak_) was simulated by adding the following equation to the set of ion currents included into the Pandit model:

*I*_*leak*_ = *G*_*leak*_. (*V*_*m*_ − *E*_*K*_), where G_leak_ is the hypothesized membrane leakage conductance and E_K_ is the potassium reversal potential.

### Genotoxicity assay

DNA damage was measured using single-cell gel electrophoresis (SCGE, Comet assay). The alkaline version (pH >13) of the assay was performed to detect single-strand breaks and alkali-labile sites, such as apyrimidinic and apurinic sites that are formed when bases are lost and oxidized. SCGE was performed basically according to [41], with some minor modifications applied to adapt the procedure to cardiomyocytes (see Additional file 1: Methods section).

### Measurement of ROS formation in single cells exposed to TiO_2_-NPs

ROS production was measured by fluorescence using 5’**-**(and 6)**-**carboxy-2’ **-**7’ -dihydrodichlorofluorescein diacetate (DCFDA) as described by [42]. See Additional file 1: Methods section.

### Multicellular studies

#### Intratracheal instillation

After anesthesia and in order to avoid forcing out of NPs into the lung, a 16-gauge catheter was gently inserted into the trachea of rats in order to deliver ~50 μl of saline solution (Vehicle, n = 6) or saline solution +2 mg/kg TiO_2_ (NP_R_, n = 8) by means of a laboratory bench P200 pipette (Gibson, UK). The volume was determined on a body weight basis in order to ensure good NP dispersion (after sonification). This amount of saline solution minimizes the dose of fluid contacting the lungs. Furthermore, an empty sterile syringe (1 ml) was used to gradually inflate the lung with air twice prior to administration of 0.15 mg/kg atipamezole hydrochloride (Antisedan, Pfizer, Italy); finally, the cannula port was connected to a ventilator (Rodent ventilator UB 7025, Ugo Basile, Comerio, Italy). We chose a single concentration of 2 mg/kg as the LD_50_ for this nanomaterial is 59.2 mg/Kg, and we wanted to operate safely (~4%) in line with recently described studies [43]. After waking, the rats were left conscious for four hours before performing *in vivo* electrophysiological recordings.

Before exposing the rat heart, pre-cordial ECGs (3 unipolar and 3 bipolar leads) were recorded in order to screen possible anoxic effects on cardiac electrical activity due to the tracheal instillation (data not shown). Two animals, one per group, were excluded from the final analysis because of the presence of anoxic signs, possibly related to mechanical injury due to the instillation procedure (atrioventricular block).

### Epicardial multiple leads recording

Four hours after the instillation, the rats were re-anesthetized. Under artificial respiration, the heart was exposed through a longitudinal sternotomy and suspended in a pericardial cradle. Body temperature was maintained with infrared lamp radiation. In the present study, an 8 × 8 row electrode matrix with 1**-**mm inter-nodal resolution was fabricated from surgical cotton gauze. The electrode array was positioned in order to cover part of the anterior surface of the right (RV) and left (LV) ventricles [44], and the following parameters acquired: ECG waves, excitability parameters (Rheobase and Chronaxie), longitudinal and transversal conduction velocities (CVl, CVt) and ERP. See Additional file 1: Methods section.

### Detection of TiO_2_ nanoparticles in heart and lungs by TEM

Heart (RV and LV) and lung samples from Vehicle and NP_R_ groups were analyzed by TEM in order to document the presence of NPs within the tissues. Moreover, freshly isolated rat cardiomyocytes were incubated with 50 μg/ml TiO_2_ and processed for ultrastructural analysis (see Additional file 1: Methods section).

### Thiobarbituric acid reactive substance detection

Tissue samples were extracted in the dark, washed in PBS and included in criovials prior to freezing at −80°C. Frozen tissue samples (trachea, lungs and heart) were homogenized and sonicated in phosphate-buffered saline supplemented with protease inhibitor cocktail (Sigma-Aldrich, St. Louis, MO, USA). Insoluble debris was pelleted and lipid peroxidation products detected in the supernatants by the TBARS method, based on the condensation of malondialdehyde derived from polyunsaturated fatty acids, with two equivalents in order to give a fluorescent red derivative. More details in the Additional file 1: Methods section.

### Statistical analysis

The SPSS statistical package was used (SPSS 17th version, Chicago, IL, USA). Normal distribution of variables was checked by means of the Kolmogorov**-**Smirnov test. Statistics of normally distributed variables included mean ± standard error (SE), paired and unpaired Student’s t test. Statistical significance was set at p < 0.05.

## Additional file

Additional file 1: **Supplementary Material.**

## Abbreviations

(TiO_2_): Titanium dioxide
(NPs): Nanoparticles
(NIOSH): National Institute of Occupation Safety and Health
(AP): Action potential
(APD): Action potential duration
(APA): Action potential amplitude
(UPS): Action potential upstroke
(ROS): Reactive oxygen species
(AFM): Atomic force microscopy
(V_m_): Transmembrane potential
(V_r_): Resting potential
(C_m_): Membrane capacitance
(R_m_): Membrane resistance
(CTRL): Control non-exposed cells
(NP_C_): TiO_2_-exposed cells
(NP_R_): TiO2-exposed rats
(TBARS): Thiobarbituric acid reactive substance
(CV): Coefficient of variability
(RV): Right ventricle
(LV): Left ventricle
(SCs): Spontaneous contractions
(CVt): Transverse conduction velocity
(CVl): Longitudinal conduction velocity
(ERP): Effective refractory period
(SCGE): Single-cell gel electrophoresis
(TEM): Transmission electron microscopy
(FS): Fraction of shortening

## References

[1] Shi H, Magaye R, Castranova V, Zhao J (2013). Titanium dioxide nanoparticles: a review of current toxicological data. Part Fibre Toxicol.

[2] Zhen S, Qian Q, Jia G, Zhang J, Chen C, Wei Y (2012). A panel study for cardiopulmonary effects produced by occupational exposure to inhalable titanium dioxide. J Occup Environ Med.

[3] Participants IRSIW (2000). The relevance of the rat lung response to particle overload for human risk assessment: a workshop consensus report. ILSI risk science institute workshop participants. Inhal Toxicol.

[4] Inoue K, Takano H, Ohnuki M, Yanagisawa R, Sakurai M, Shimada A, Mizushima K, Yoshikawa T (2008). Size effects of nanomaterials on lung inflammation and coagulatory disturbance. Int J Immunopathol Pharmacol.

[5] Yazdi AS, Guarda G, Riteau N, Drexler SK, Tardivel A, Couillin I, Tschopp J (2010). Nanoparticles activate the NLR pyrin domain containing 3 (Nlrp3) inflammasome and cause pulmonary inflammation through release of IL-1alpha and IL-1beta. Proc Natl Acad Sci U S A.

[6] Ferin J, Oberdorster G, Penney DP (1992). Pulmonary retention of ultrafine and fine particles in rats. Am J Respir Cell Mol Biol.

[7] Sager TM, Kommineni C, Castranova V (2008). Pulmonary response to intratracheal instillation of ultrafine versus fine titanium dioxide: role of particle surface area. Part Fibre Toxicol.

[8] Lee KP, Henry NW, Trochimowicz HJ, Reinhardt CF (1986). Pulmonary response to impaired lung clearance in rats following excessive TiO2 dust deposition. Environ Res.

[9] Lee KP, Gillies PJ (1986). Pulmonary response and intrapulmonary lipids in rats exposed to bismuth orthovanadate dust by inhalation. Environ Res.

[10] Choi HS, Ashitate Y, Lee JH, Kim SH, Matsui A, Insin N, Bawendi MG, Semmler-Behnke M, Frangioni JV, Tsuda A (2010). Rapid translocation of nanoparticles from the lung airspaces to the body. Nat Biotechnol.

[11] Link MS, Luttmann-Gibson H, Schwartz J, Mittleman MA, Wessler B, Gold DR, Dockery DW, Laden F (2013). Acute exposure to air pollution triggers atrial fibrillation. J Am Coll Cardiol.

[12] Helfenstein M, Miragoli M, Rohr S, Muller L, Wick P, Mohr M, Gehr P, Rothen-Rutishauser B (2008). Effects of combustion-derived ultrafine particles and manufactured nanoparticles on heart cells in vitro. Toxicology.

[13] Bersani D, Lottici PP, Ding XZ (1998). Phonon confinement effects in the Raman scattering by TiO2 nanocrystals. Appl Phys Lett.

[14] Djaoued Y, Badilescu S, Ashrit PV, Bersani D, Lottici PP, Robichaud J (2002). Study of anatase to rutile phase transition in nanocrystalline titania films. J Sol-Gel Sci Technol.

[15] Zaniboni M, Cacciani F, Salvarani N (2007). Temporal variability of repolarization in rat ventricular myocytes paced with time-varying frequencies. Exp Physiol.

[16] Pandit SV, Clark RB, Giles WR, Demir SS (2001). A mathematical model of action potential heterogeneity in adult rat left ventricular myocytes. Biophys J.

[17] Rohr S, Kucera JP, Kleber AG (1998). Slow conduction in cardiac tissue, I: effects of a reduction of excitability versus a reduction of electrical coupling on microconduction. Circ Res.

[18] Gutstein DE, Morley GE, Tamaddon H, Vaidya D, Schneider MD, Chen J, Chien KR, Stuhlmann H, Fishman GI (2001). Conduction slowing and sudden arrhythmic death in mice with cardiac-restricted inactivation of connexin43. Circ Res.

[19] Laurita KR, Girouard SD, Akar FG, Rosenbaum DS (1998). Modulated dispersion explains changes in arrhythmia vulnerability during premature stimulation of the heart. Circulation.

[20] Roell W, Lewalter T, Sasse P, Tallini YN, Choi BR, Breitbach M, Doran R, Becher UM, Hwang SM, Bostani T, von Maltzahn J, Hofmann A, Reining S, Eiberger B, Gabris B, Pfeifer A, Welz A, Willecke K, Salama G, Schrickel JW, Kotlikoff MI, Fleischmann BK (2007). Engraftment of connexin 43-expressing cells prevents post-infarct arrhythmia. Nature.

[21] Miragoli M, Novak P, Ruenraroengsak P, Shevchuk AI, Korchev YE, Lab MJ, Tetley TD, Gorelik J (2013). Functional interaction between charged nanoparticles and cardiac tissue: a new paradigm for cardiac arrhythmia?. Nanomedicine (Lond).

[22] Novak P, Shevchuk A, Ruenraroengsak P, Miragoli M, Thorley AJ, Klenerman D, Lab MJ, Tetley TD, Gorelik J, Korchev YE (2014). Imaging single nanoparticle interactions with human lung cells using fast ion conductance microscopy. Nano Lett.

[23] Hyder MN, Gallant BM, Shah NJ, Shao-Horn Y, Hammond PT (2013). Synthesis of highly stable sub-8 nm TiO2 nanoparticles and their multilayer electrodes of TiO2/MWNT for electrochemical applications. Nano Lett.

[24] El-Negoly SA, El-Fallal AA, El-Sherbiny IM (2014). A New modification for improving shear bond strength and other mechanical properties of conventional glass-ionomer restorative materials. J Adhes Dent.

[25] Xu J, Shi H, Ruth M, Yu H, Lazar L, Zou B, Yang C, Wu A, Zhao J (2013). Acute toxicity of intravenously administered titanium dioxide nanoparticles in mice. PLoS One.

[26] Leroy P, Tournassat C, Bizi M (2011). Influence of surface conductivity on the apparent zeta potential of TiO2 nanoparticles. J Colloid Interface Sci.

[27] Chen T, Hu J, Chen C, Pu J, Cui X, Jia G (2013). Cardiovascular effects of pulmonary exposure to titanium dioxide nanoparticles in ApoE knockout mice. J Nanosci Nanotechnol.

[28] de Lange E, Kucera JP (2010). Alternans resonance and propagation block during supernormal conduction in cardiac tissue with decreased [K(+)](o). Biophys J.

[29] Rohr S, Kucera JP (1997). Involvement of the calcium inward current in cardiac impulse propagation: induction of unidirectional conduction block by nifedipine and reversal by Bay K 8644. Biophys J.

[30] Patel C, Yan GX, Antzelevitch C (2010). Short QT syndrome: from bench to bedside. Circ Arrhythmia Electrophysiol.

[31] Kim JB, Kim C, Choi E, Park S, Park H, Pak HN, Lee MH, Shin DC, Hwang KC, Joung B (2012). Particulate air pollution induces arrhythmia via oxidative stress and calcium calmodulin kinase II activation. Toxicol Appl Pharmacol.

[32] Stilli D, Aimi B, Sgoifo A, Ciarlini P, Regoliosi G, Lagrasta C, Olivetti G, Musso E (1998). Dependence of temporal variability of ventricular recovery on myocardial fibrosis. Role of mechanoelectric feedback?. Cardiovasc Res.

[33] Sachse FB, Torres NS, Savio-Galimberti E, Aiba T, Kass DA, Tomaselli GF, Bridge JH (2012). Subcellular structures and function of myocytes impaired during heart failure are restored by cardiac resynchronization therapy. Circ Res.

[34] Stampfl A, Maier M, Radykewicz R, Reitmeir P, Gottlicher M, Niessner R (2011). Langendorff heart: a model system to study cardiovascular effects of engineered nanoparticles. ACS Nano.

[35] Sheng L, Wang X, Sang X, Ze Y, Zhao X, Liu D, Gui S, Sun Q, Cheng J, Cheng Z, Hu R, Wang L, Hong F (2013). Cardiac oxidative damage in mice following exposure to nanoparticulate titanium dioxide. J Biomed Mater Res A.

[36] Driscoll KE, Lindenschmidt RC, Maurer JK, Higgins JM, Ridder G (1990). Pulmonary response to silica or titanium dioxide: inflammatory cells, alveolar macrophage-derived cytokines, and histopathology. Am J Respir Cell Mol Biol.

[37] Boyden PA, Davies SS, Viswanathan PC, Amarnath V, Balser JR, Roberts LJ (2007). Potential role of isoketals formed via the isoprostane pathway of lipid peroxidation in ischemic arrhythmias. J Cardiovasc Pharmacol.

[38] Bihari P, Vippola M, Schultes S, Praetner M, Khandoga AG, Reichel CA, Coester C, Tuomi T, Rehberg M, Krombach F (2008). Optimized dispersion of nanoparticles for biological in vitro and in vivo studies. Part Fibre Toxicol.

[39] Berni R, Savi M, Bocchi L, Delucchi F, Musso E, Chaponnier C, Gabbiani G, Clement S, Stilli D (2009). Modulation of actin isoform expression before the transition from experimental compensated pressure-overload cardiac hypertrophy to decompensation. Am J Physiol Heart Circ Physiol.

[40] Zaniboni M, Cacciani F, Groppi M (2005). Effect of input resistance voltage-dependency on DC estimate of membrane capacitance in cardiac myocytes. Biophys J.

[41] Singh NP, McCoy MT, Tice RR, Schneider EL (1988). A simple technique for quantitation of low levels of DNA damage in individual cells. Exp Cell Res.

[42] Giordano G, Afsharinejad Z, Guizzetti M, Vitalone A, Kavanagh TJ, Costa LG (2007). Organophosphorus insecticides chlorpyrifos and diazinon and oxidative stress in neuronal cells in a genetic model of glutathione deficiency. Toxicol Appl Pharmacol.

[43] Tassinari R, Cubadda F, Moracci G, Aureli F, D'Amato M, Valeri M, De Berardis B, Raggi A, Mantovani A, Passeri D, Rossi M, Maranghi F (2014). Oral, short-term exposure to titanium dioxide nanoparticles in Sprague–Dawley rat: focus on reproductive and endocrine systems and spleen. Nanotoxicology.

[44] Rossi S, Baruffi S, Bertuzzi A, Miragoli M, Corradi D, Maestri R, Alinovi R, Mutti A, Musso E, Sgoifo A, Brisinda D, Fenici R, Macchi E (2008). Ventricular activation is impaired in aged rat hearts. Am J Physiol Heart Circ Physiol.

