# Supplementary material for: Titanium dioxide nanoparticles promote arrhythmias via a direct interaction with rat cardiac tissue
Source: Part Fibre Toxicol. 2014 Dec 9;11:63. doi: 10.1186/s12989-014-0063-3 (PMC4349471; doi:10.1186/s12989-014-0063-3)
Supplement: Supplementary file 1 — Supplementary Material. [file 12989_2014_63_MOESM1_ESM.zip › Supplemental Figures.pdf]

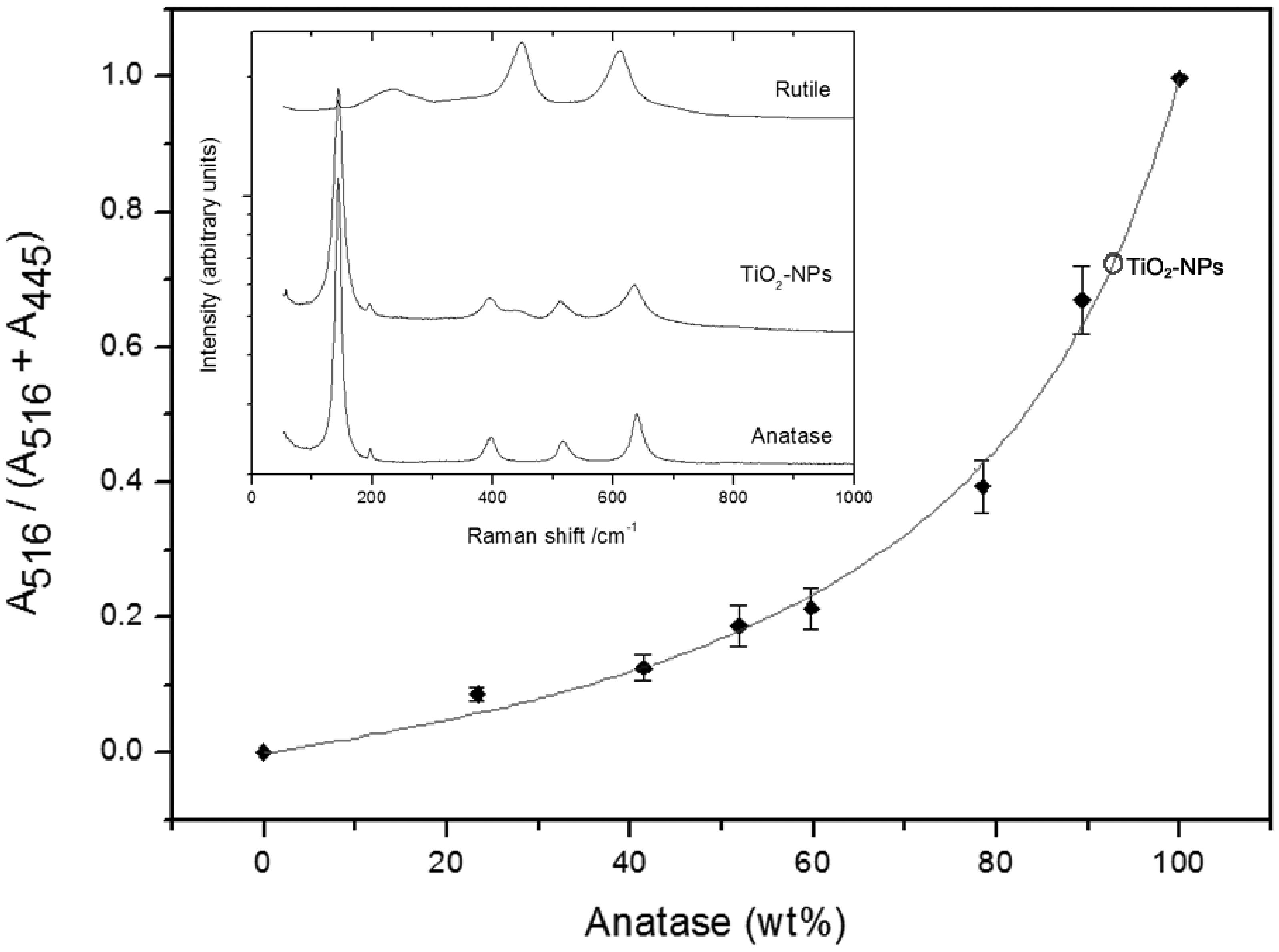

Rutile
TiO2-NPs
Anatase
Intensity (arbitrary units)
0
200
400
600
800
1000
Raman shift /cm-1
TiO2-NPs
1.0
0.8
0.6
0.4
0.2
0.0
A516 / (A516 + A445)
0
20
40
60
80
100
Anatase (wt%)

Figure S1

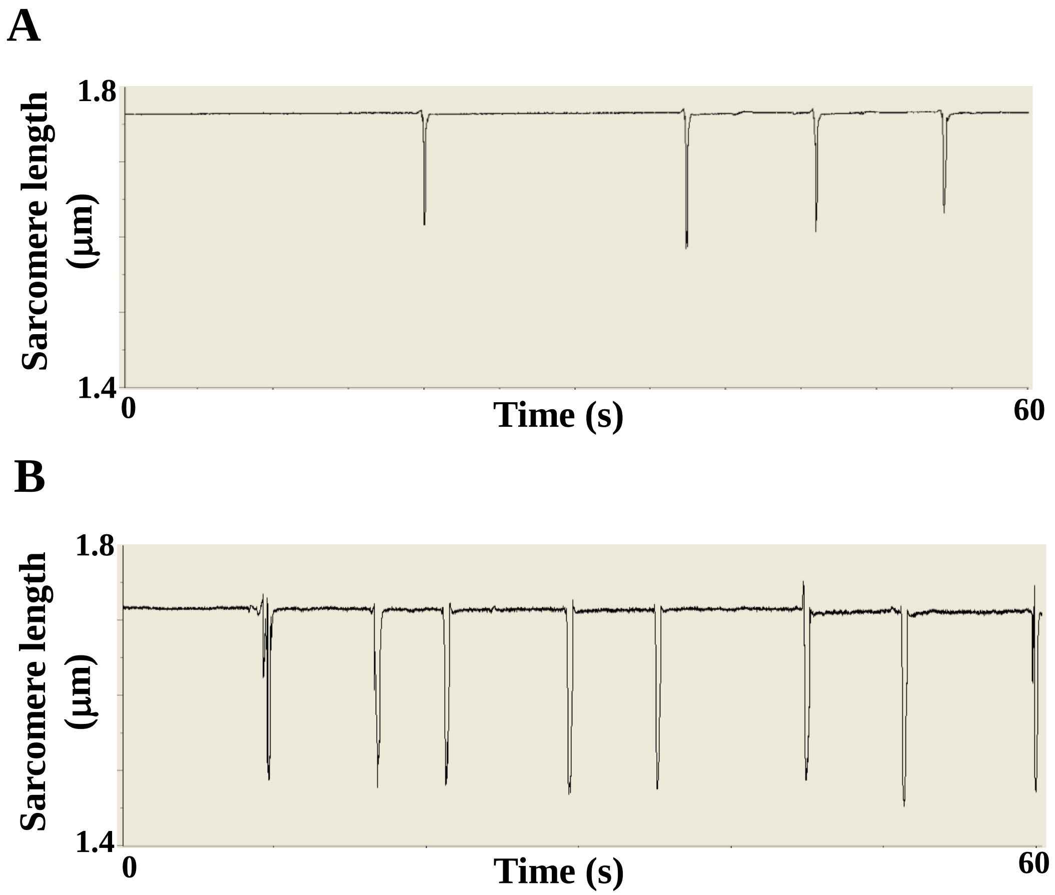
A
1.8
Sarcomere length (µm)
1.4
0
Time (s)
60
B
1.8
Sarcomere length (µm)
1.4
0
Time (s)
60

Figure S2

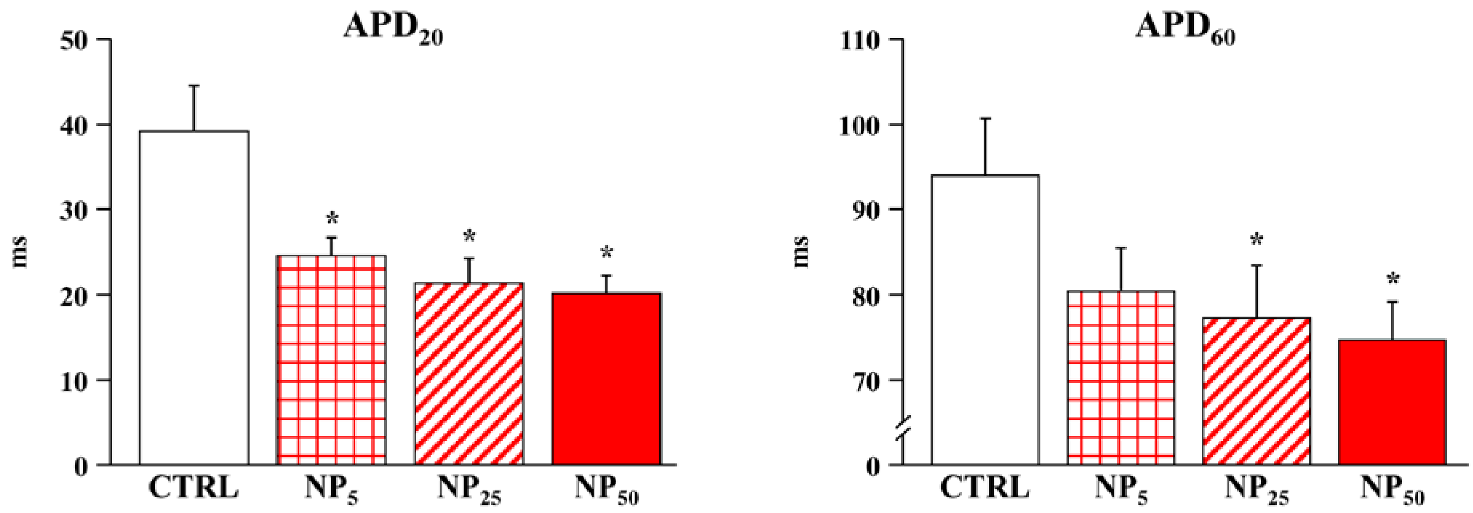

APD20
50
40
30
20
10
0
ms
*
*
*
CTRL
NP5
NP25
NP50
APD60
110
100
90
80
70
0
ms
*
*
CTRL
NP5
NP25
NP50

Figure S3

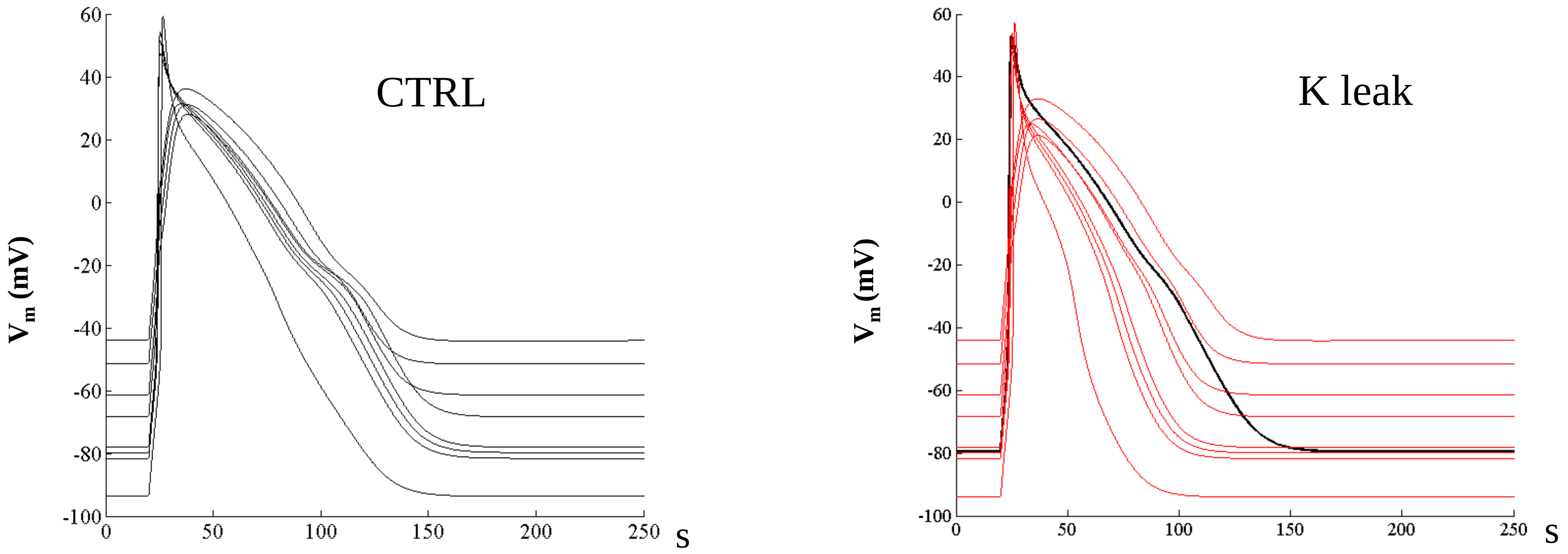

$[K^+]_o$ (mmol/l)	V_r CTRL (mV)	V_r K leak (mV)
**3.0**	**-93.7**	**-94.00**
**5.0**	**-81.6**	**-81.78**
**5.4**	**-79.77**	**-79.93**
**5.8**	**-78.06**	**-78.21**
**8.7**	**-68.37**	**-68.44**
**11.6**	**-61.43**	**-61.46**
**17.4**	**-51.44**	**-51.44**
**23.2**	**-44.05**	**-44.03**

Figure S4

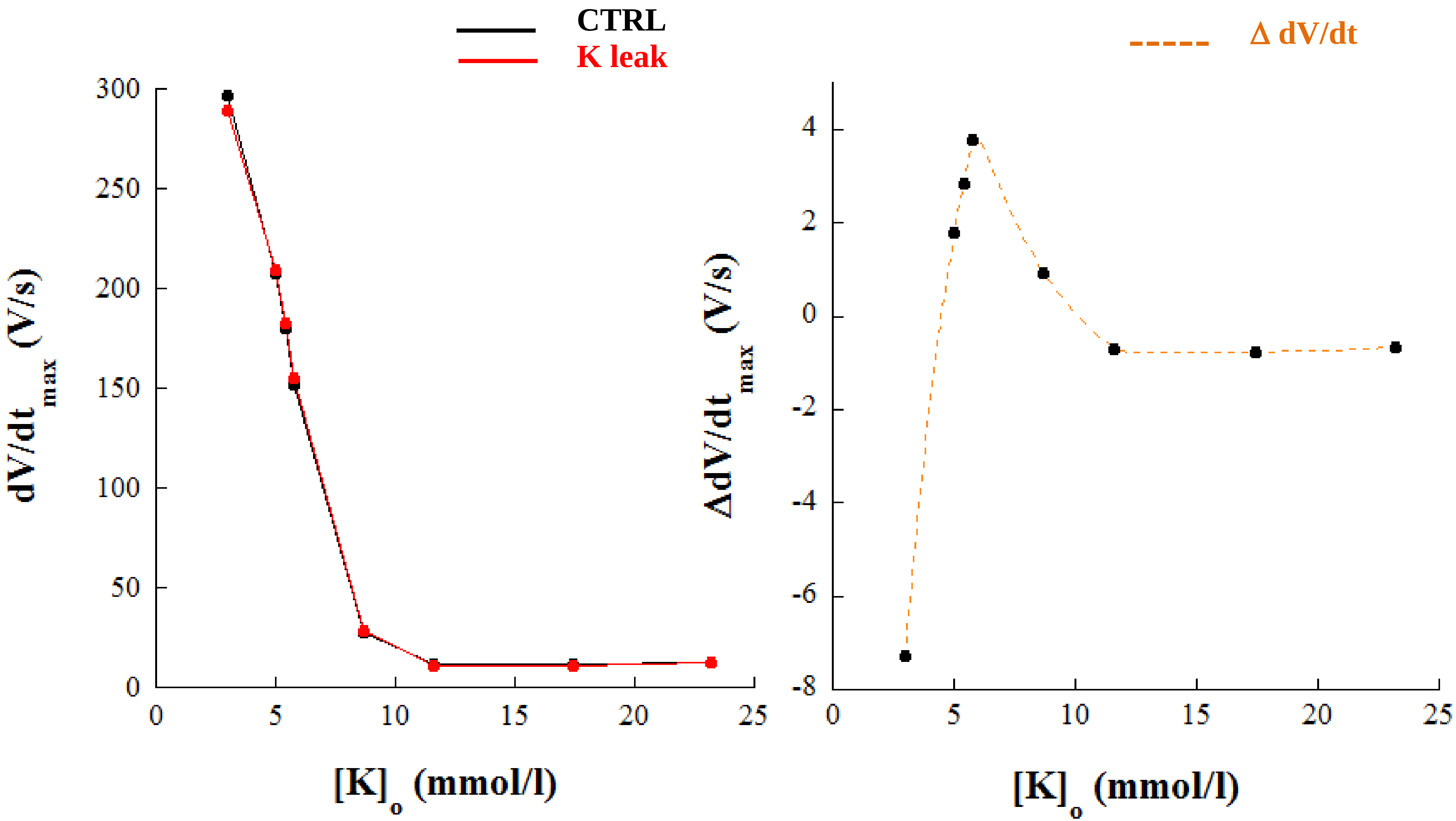
CTRL
K leak
Δ dV/dt
300
250
200
150
100
50
0
4
2
0
-2
-4
-6
-8
0
5
10
15
20
25
dV/dt max (V/s)
ΔdV/dt max (V/s)
[K]o (mmol/l)

Figure S5

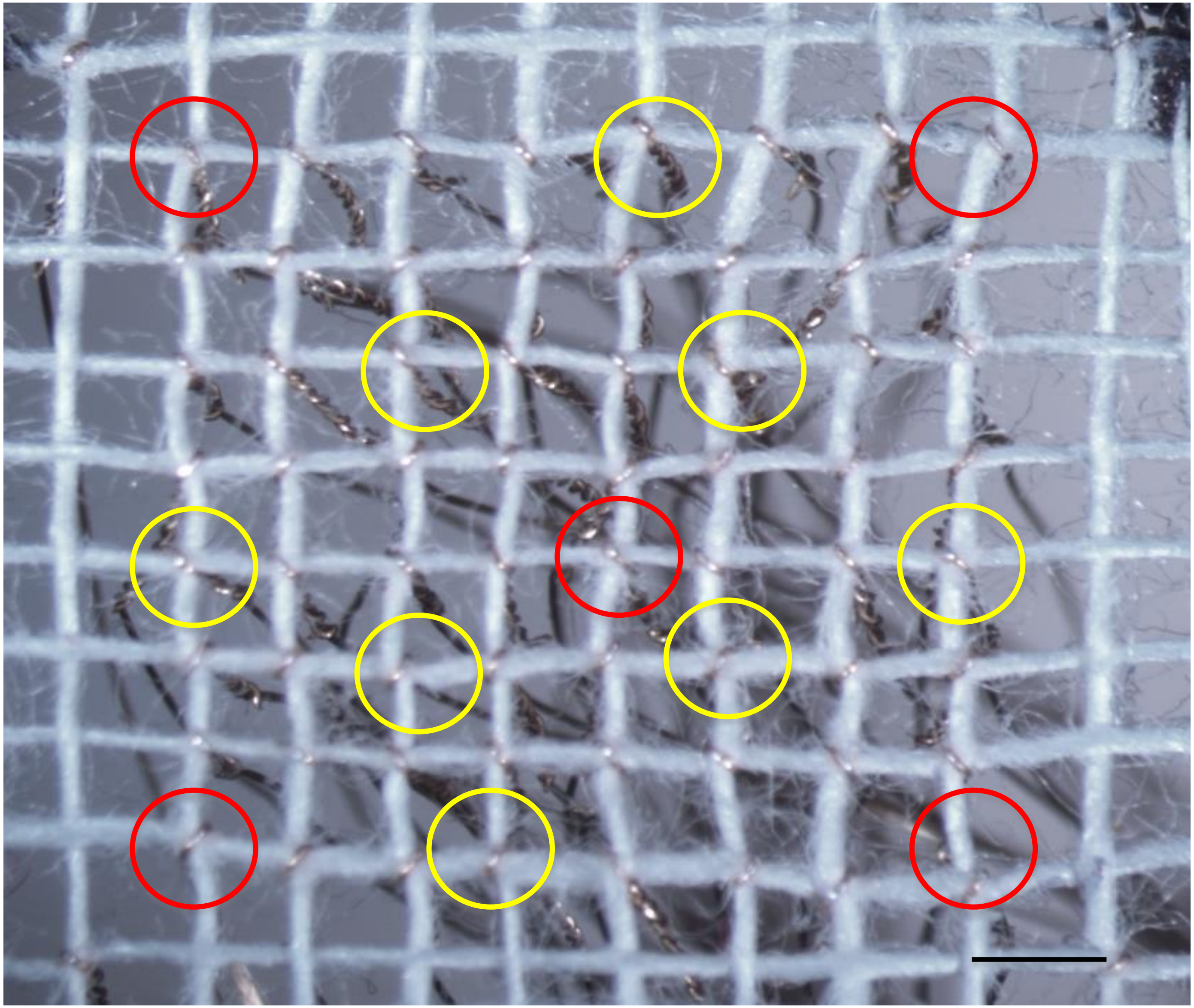

Figure S6

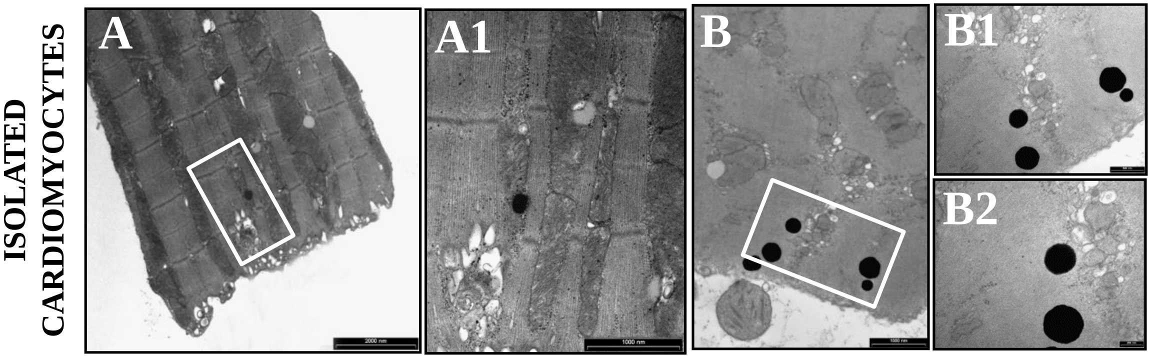

Figure S7

	CTRL	NP_C
**R_m (MΩ)**	36.4 ± 1.98	38.01 ± 1.93
**V_m (mV)**	-71.72 ± 0.58	-70.93 ± 0.41
**APA (mV)**	80.5 ± 1.12	77.9 ± 1.21
**Rheobase (pA/pF)**	0.0076 ± 0.001	0.0088 ± 0.001
**Chronaxie (ms)**	1.68 ± 0.055	1.69 ± 0.100

Table S1
