# Supplementary material for: Titanium dioxide nanoparticles promote arrhythmias via a direct interaction with rat cardiac tissue
Source: Part Fibre Toxicol. 2014 Dec 9;11:63. doi: 10.1186/s12989-014-0063-3 (PMC4349471; doi:10.1186/s12989-014-0063-3)
Supplement: Supplementary file 1 — Supplementary Material. [file 12989_2014_63_MOESM1_ESM.zip › Supplemental Material Section.pdf]

Supplemental Material

Titanium dioxide nanoparticles promote arrhythmias via a direct interaction with rat cardiac tissue

Supplemental Methods

AFM imaging processing

AFM analysis showed that the deposition of 50µg/ml NPs solution on poly-ornithine treated mica, which changes the surface charge from negative to positive[1], leads to an increases of NPs adhesion (data not shown). The average volume of NPs with a height less than 100 nm was used to calculate the total amount of NPs in each aggregate. By taking into account the dead volume and the tip broadening effects[2], the computed number of NPs, present in each aggregate was divided by a factor of two. Thus, the ratio between the number of NPs with a height less than 100 nm and the total number of NPs (single or aggregated) was used to calculate the frequency of single NPs.

Determination of anatase and rutile proportion by Raman spectroscopy

It's well known that commercial TiO_2-NPs products are a mixture of different TiO_2 polymorphs (mainly anatase and rutile, occasionally brookite). Raman spectroscopy is a powerful technique to discriminate between polymorphs. Titanium dioxide exhibits very high Raman efficiency and therefore a measurement of the amount of the different phases has been performed on the TiO_2-NPs powder samples. A calibration curve to determine the anatase amount in the TiO_2-NPs powder has been created by collecting Raman spectra on mixtures of anatase and rutile laboratory references (XRD tested), at nine different compositions ranging from 100 wt% anatase to 100 wt% rutile. Different ratios R of the areas (A) of selected anatase and rutile Raman bands ($R_{516/445}$= A_{516} /

$(A_{516} + A_{445})$; $R_{143/445}= A_{143} / (A_{141} + A_{445})$; $R_{143/609}= A_{143} / (A_{141} + A_{609})$; $R_{516/609}= A_{516} / (A_{516} + A_{609})$ have been determined by a peak-fitting procedure, as a function of the anatase content x (wt%). For each pair of Raman bands, the function $y=ax/(ax+100-x)$ was used to fit the data, a being a fitting parameter, taken as the ratio of the Raman absolute intensities of anatase and rutile selected bands. The fitting curves, with a goodness of fit parameter R^2 ~ 0.99, (see the fit to $A_{516} / (A_{516} + A_{445})$in Fig.S1) have then been used to determine the x value for the TiO_2-NPs in the commercial product.

Cardiomyocytes isolation

After rat sacrifice, the heart was rapidly removed, mounted on a Langerdorff apparatus and perfused at 37°C with the following sequence of solutions: 1) a calcium-free solution (stock 1) for 5 min containing the following (in mmol/l): 126 NaCl, 22 dextrose, 5.0 $MgCl_2$, 4.4 KCl, 20 taurine, 5 creatine, 5 Na pyruvate, 1 NaH_2PO_4, and 24 HEPES (pH = 7.4, adjusted with NaOH); 2) stock 1 plus 0.1mmol/l Ca^{2+}, 1mg/ml type 2 collagenase (Worthington Biochemical, USA), and 0.1mg/ml type XIV protease (Sigma, Milan, Italy) for about 20 min; and 3) solution 1 plus 0.1mmol/l Ca^{2+} (enzyme-free) for 5 min. All solutions were gassed with 100% O_2. The LV was then minced and shaken for 10 min. Cells were filtered through a nylon mesh and re-suspended in low-calcium solutions: 0.1mmol/l (for 30 min) and 0.5mmol/l (maintenance solution, additional 30 min). Sonicated solutions containing TiO_2-NPs were eventually added to the myocyte suspension at the concentration of 50μg/ml.

After one hour of exposure, cardiomyocytes were placed in a chamber mounted on the stage of an inverted microscope (Nikon-Eclipse TE2000-U, Nikon Instruments, Japan) and superfused (≈1ml/min at 37°C) with a Tyrode solution containing (in mmol/l): 140 NaCl, 5.4 KCl, 1 $MgCl_2$, 5 HEPES, 5.5 glucose, and 1.8 $CaCl_2$ (pH = 7.4, adjusted with NaOH). Myocyte mechanical and electrical properties were then determined both in

control (CTRL) and treated (NP_C) cells. Oxidative stress and genotoxicity were also studied in the same experimental conditions.

All experiments were performed within 5 hours after isolation. All myocytes used were rod-shaped, had well-defined striations with an average sarcomere length of 1.7 μm.

Cell Contractility

Mechanical properties of ventricular cardiomyocytes were assessed by using the IonOptix fluorescence and contractility systems (IonOptix, Milton, MA, USA). Cells were perfused with the Tyrode solution and field stimulated at a frequency of 0.5, 1 and 2Hz by constant depolarizing pulses. Load-free contractions of cardiomyocytes were measured as average sarcomere shortening through a fast Fourier transform algorithm. In 206 cardiomyocytes (104 CTRL and 102 NP_C). Steady-state contraction of cardiomyocytes was achieved before data recording by means of conditioning electrical stimulation trains.

Patch-clamp Technique

Suction pipettes were made from borosilicate capillary tubing (Harvard Apparatus LTD, Edenbridge, UK) with an access resistance of 2 to 4 MΩ when filled. Normal pipette filling solution contained the following (in mmol/l): 113 KCl, 10 NaCl, 5.5 dextrose, 5 K_2ATP, 0.5 $MgCl_2$, and 10 HEPES (pH = 7.1 adjusted with KOH). Membrane capacitance (C_m) and resistance (R_m) were derived according to a protocol previously described[3] by means of 100 ms hyperpolarizing constant current pulses.

Time was allowed for AP waveforms to reach a steady state configuration (usually within 30 beats). Then, 10 consecutive APs were recorded and averaged and the following parameters were measured: i) AP upstroke (UPS, mV) as the maximum value reached by V_m in the initial phase of AP, ii) action potential amplitude (APA, mV) as the difference between UPS and the preceding V_r and iii) AP duration at -20 mV

(APD_{20}) and -60 mV (APD_{60}), respectively corresponding to the early and late phase of repolarization. The APD was calculated as the interval between the time of maximal upstroke velocity (dV/d_{tmax}) and the time when V_m reached -20 and -60 mV. Beat-to-beat variability, measured at a repolarization potential of -60 mV, was derived as the coefficient of variability of APD of the10 consecutive APs (CV_{APD60})[4].

Finally, cell excitability was evaluated by strength–duration (S-D) curves, i.e. lowest (threshold) current required for eliciting an AP, reported versus pulse duration. To this aim, single cardiomyocytes were conditionally paced with ten supra-threshold current pulses (1 ms in duration) at 5 Hz; the stimulus duration was suddenly changed (range: 5ms - 0.3ms) and the corresponding current threshold measured.

The current threshold found at 5ms was taken as the rheobase (Rh). Each Rh value was normalized to the corresponding C_m (pA/pF). Chronaxie (Chr) was taken as the stimulus duration needed to elicit an AP when the current strength was set twice the Rh.

The S-D curve is represented by an hyperbolic equation: where I = current strength and T = pulse duration:

$$I=Rh(1+Chr/T)$$

Both Rh and Chr characterize tissue excitability.

Genotoxicity assay

Immediately after enzymatic isolation, cardiomyocytes were treated with 50 μg/ml TiO_2 NPs suspension using. After incubation, cells were centrifuged (5 min, 800g) to remove nanoparticles containing medium. Cell pellets were resuspended in 1 ml of HBSS diluted 1:9 and incubated at RT for 30 min in order to prepare cardiomyocytes for the lysis before the last centrifugation (1 min, 800g), which was needed to obtain a reasonable amount of cell pellets. The pellets were then resuspended in 90 mL Low

Melting Agarose 0.7% (LMA), transferred onto degreased microscope slides previously dipped in 1% Normal Melting Agarose (NMA) for the first layer. The agarose was allowed to set for 15 min at 4°C before addition of a final layer of low melting agarose (LMA). Cell lysis was carried out at 4°C overnight by exposing cells to a buffer containing 2.5M NaCl, 100mmol/l Na_2EDTA, 8mmol/l Tris-HCl, 1% Triton X-100 and 10% DMSO, pH=10. The electrophoretic migration was performed (DNA unwinding: 20 min; electrophoresis: 20min, 0.78Vcm^{-1}, 300mA) nn an alkaline buffer, pH>13, (1mmol/l Na_2EDTA, 300mmol/l NaOH, 0°C).

DNA was stained with 75µL ethidium bromide (10µg/ml) before the examination at 400X magnification under a Leica DMLS fluorescence microscope (excitation filter BP 515 - 560nm, barrier filter LP 580nm), using an automatic image analysis system (Comet Assay III– Perceptive Instruments Ltd, UK).

Total percentage of fluorescence in DNA fragmentation tail (TI, tail intensity) provided representative data on the genotoxic effects. For each sample, coded and evaluated blind, 100 cells were analyzed. All steps of the comet assay were conducted under yellow light.

ROS detection in-vitro

Upon entering cells DCFDA is de-esterificated and then oxidized by ROS to its fluorescent form. In a typical experiment, freshly extracted cardiomyocytes were washed with PBS and then pre-incubated for 30 min (37 °C) with DCFDA (20 µmol/L), which was added from a stock solution in DMSO and diluted in PBS. The quantity of DMSO never exceeded 0.1%, and was also added to the blank. Cells were washed with HBSS to remove extracellular DCFDA. After treatments (3 h at 37°C), incubation medium was removed and a solution of Tris-HCl-TritonX and a cell dissociation solution (Sigma, Milan, Italy) were added for 10 min. After

centrifugation the supernatant was collected, and the fluorescence was immediately read with a fluorescence spectrophotometer (Cary Eclipse, Varian, Palo Alto, CA, USA) looking at the fluorescence peak between 510 and 550 nm (excitation = 480 nm).

In-vivo cardiac electrophysiological measurement

In each experiment, unipolar epicardial electrograms (EGs) were recorded from each electrode within the epicardial array and a common reference electrode placed on the left hind leg, during normal sinus rhythm and ventricular pacing. Hearts were paced by near-threshold, ≤ 1 ms duration, cathode current pulses at a frequency slightly higher than spontaneous rhythm (≈250 bpm). During pacing, unipolar EGs were recorded from all the electrodes except at the paced site.

The following measurements were carried out:

EGs waves and intervals durations, refractoriness and inducible ectopic activity, ventricular excitability and conduction velocities.

Interval duration. The duration of R-R interval, P wave, PQ segment, and QRS complex was measured from the root mean square signal computed from the 8×8 EGs (RMS). QT duration was computed as the interval between QRS onset and the time of the minimum of the first derivative of RMS signal, during the descending limb of T-wave.

Refractoriness and induced arrhythmia. We delivered eight consecutive basic stimuli (S1), 1ms long and with an amplitude twice diastolic threshold through 8 electrodes randomly chosen within the array (Figure S6). The S1 pacing sequence was followed by an extra stimulus (S2) whose delay from previous S1 was first progressively decremented by 10 ms steps until capture was lost and then progressively incremented by 2 ms steps until the capture was resumed. The longest S1-S2 coupling interval that

did not originate ventricular capture identified the effective refractory period (ERP) at a given electrode site, enabling the characterization of spatial differences in tissue refractoriness.

Ventricular Excitability. Cardiac excitability was measured by strength-duration relationships derived from five electrodes of the grid from each animal as previously described[5]. From such relationship, we extrapolated Rheobase and Chronaxie parameters; the former parameter indicates the minimal magnitude of depolarizing current needed at nominally infinite stimulus duration in order to elicit an active response, while the latter is the stimulus duration needed to reach the threshold for activation by using a current amplitude twice the Rheobase.. Five electrodes of the grid (the four corners and a central one, Figure S6) were initially paced with current pulses of decreasing strength (starting from supra-threshold values) at a constant duration of 8 ms until activation failed, thus identifying the threshold current. The procedure was repeated with current pulses of progressively shorter duration, down to 0.01 ms.

Conduction velocity. Activation times were estimated using the instant of the minimum time derivative of unipolar EGs during QRS, and were referenced to the stimulus onset. From the activation times of paced beats, an activation sequence (isochrone map) was determined where Conduction Velocity (CV) was computed longitudinally (CVl) and transversally (CVt) to fiber orientation. Briefly, CVl was evaluated from electrodes distant from the pacing site on the major axis of the elliptical wave front; CVt was evaluated from electrodes on a line perpendicular to the major axis of the elliptical wave front across the more closely spaced isochrones. It is known that the long axis of the elliptical wave-front is parallel to the local fiber direction at the pacing site[6]. The anisotropy ratio was determined as CVl/CVt.

TBARS detections ex-vivo

300µl of tissue supernatant were mixed with an equal volume of 0.2 mol orthophosphoric acid and a 1/16 volume of thiobarbituric acid in 0.1M NaOH and incubated for 45 minutes at 90° C. TBARS were extracted with n-butanol/NaCl and, after centrifugation, fluorescence was measured using a Cary Eclipse fluorescence spectrophotometer (excitation 515 nm, emission 545 nm). Malondialdehyde was used as a standard for the calibration curve. TBARS concentrations were normalized to total protein concentration in each sample, determined by the BCA (bicinchoninic acid) Protein Assay (Thermo Scientific, Rockford, IL, USA).

TiO2 detection in lungs and heart by TEM

Samples were fixed in Karnovsky solution (4% formaldehyde, 5% glutaraldehyde) for 6 hours at room temperature. After washing several times with 0.1M phosphate buffer, pH 7.2, the tissue were post-fixed in 1% osmium tetroxide (OsO_4) for 90 minutes at room temperature and dehydrated by increasing concentration of alcohol. Then, samples were washed with propylene oxide and embedded in epoxy resin. Sections of 0.5 µm thickness were stained with methylene blue and safranin to morphologically select the field of interest. Subsequently, ultrathin sections of 60-80 nm thicknesses were collected on a 300-mesh copper grid and, after staining with uranyl acetate and lead citrate, were qualitatively examined under a transmission electron microscope (Philips EM 208S).

Supplementary Figure Legends

Figure S1

Calibration curve for the determination of the anatase amount in the TiO_2-NPs powder. In the inset, the Raman spectrum collected on TiO_2-NPs is compared with the Raman spectra of anatase and rutile.

Figure S2

Representative traces (60 s long) of spontaneous contraction (SCs) in both CTRL (**A**) and NP_C (**B**) isolated cardiomyocytes.

Figure S3

Dose-response characterization of NPs-induced APD shortening in acutely exposed single isolated ventricular cardiomyocytes. 5, 25 and 50 µg/ml were tested. * $p<0.05$ with respect to control.

Figure S4

Simulated APs in both CTRL and NP_C (K^+ leak) conditions at different $[K^+]_o$. Top: Simulated action potentials shape in CTRL (left, black traces) and in K^+ leak condition (right, red traces) at several $[K^+]_o$ ranging from 3 to 23.2 mmol/l. The black trace in K^+ leak APs show the AP without leakage at $[K^+]_o$ = 5.8 mmol/l. Bottom: V_r calculation in both conditions; to note, NP-like (with K^+ leak) induce a slight increment in V_r in all $[K^+]_o$ conditions up to $[K^+]_o$ = 17.4 mmol/l.

Figure S5

Upstroke velocity (dV/dt_{max}) calculated in the conditions described in (Figure S4). While dV/dt_{max} with respect to $[K^+]_o$ in CTRL and K^+ leak shows the same behavior (left), the $\Delta dV/dt_{max}$ (dV/dt_{max} NP-like - dV/dt_{max} CTRL, right) shows maximal increments around the supernormal conduction for Na current i.e. $[K^+]_o$ ~ 6 mmol/l.

Figure S6

Representative image of the 8x8 epicardial electrode array. Red circles indicate the electrodes utilized for cardiac excitability measurement. Yellow circles indicate the electrodes utilized for the ERP measurement. Scale bar: 1 mm.

Figure S7

Ultrathin sections of isolated cardiomyocyte incubated with TiO_2-NPs. **A.** The white rectangle inscribes an area shown at higher magnification in **A1**, with single NPs between mitochondria and myofibrils. **B.** Several electron-dense NPs are detected in the cytoplasm. In **B1** and **B2** the sharp profile of NPs is appreciated at higher magnification. Scale Bars: A= 2 μm; A1= 1 μm; B= 1 μm; B1= 500 nm; B2= 200 nm.

Supplemental References

1. Podesta A, Imperadori L, Colnaghi W, Finzi L, Milani P, Dunlap D: **Atomic force microscopy study of DNA deposited on poly L-ornithine-coated mica.** *J Microsc* 2004, **215:**236-240.
2. Rivetti C: **DNA contour length measurements as a tool for the structural analysis of DNA and nucleoprotein complexes.** *Methods Mol Biol* 2011, **749:**235-254.
3. Zaniboni M, Cacciani F, Groppi M: **Effect of input resistance voltage-dependency on DC estimate of membrane capacitance in cardiac myocytes.** *Biophys J* 2005, **89:**2170-2181.
4. Zaniboni M, Cacciani F, Salvarani N: **Temporal variability of repolarization in rat ventricular myocytes paced with time-varying frequencies.** *Exp Physiol* 2007, **92:**859-869.

5. Rossi S, Baruffi S, Bertuzzi A, Miragoli M, Corradi D, Maestri R, Alinovi R, Mutti A, Musso E, Sgoifo A, et al: **Ventricular activation is impaired in aged rat hearts.** *Am J Physiol Heart Circ Physiol* 2008, **295:**H2336-2347.
6. Bocchi L, Savi M, Graiani G, Rossi S, Agnetti A, Stillitano F, Lagrasta C, Baruffi S, Berni R, Frati C, et al: **Growth factor-induced mobilization of cardiac progenitor cells reduces the risk of arrhythmias, in a rat model of chronic myocardial infarction.** *PLoS One* 2011, **6:**e17750.
